# On the reaction–diffusion type modelling of the self-propelled object motion

**DOI:** 10.1038/s41598-023-39395-w

**Published:** 2023-08-03

**Authors:** Masaharu Nagayama, Harunori Monobe, Koya Sakakibara, Ken-Ichi Nakamura, Yasuaki Kobayashi, Hiroyuki Kitahata

**Affiliations:** 1 Research Center of Mathematics for Social Creativity, Research Institute for Electronic Science, Hokkaido University, Hokkaido, 060-0812 Japan; 2 Department of Mathematics, Graduate School of Science, Osaka Metropolitan University, Osaka, 599-8531 Japan; 3 Institute of Science and Engineering, Kanazawa University, Ishikawa, 920-1192 Japan; 4 RIKEN iTHEMS, Saitama, 351-0198 Japan; 5 Meiji Institute for Advanced Study of Mathematical Sciences, Meiji University, Tokyo, 164−8525 Japan; 6 Department of Physics, Chiba University, Chiba, 263-8522 Japan

**Keywords:** Applied mathematics, Nonlinear phenomena, Computational science

## Abstract

In this study, we propose a mathematical model of self-propelled objects based on the Allen–Cahn type phase-field equation. We combine it with the equation for the concentration of surfactant used in previous studies to construct a model that can handle self-propelled object motion with shape change. A distinctive feature of our mathematical model is that it can represent both deformable self-propelled objects, such as droplets, and solid objects, such as camphor disks, by controlling a single parameter. Furthermore, we demonstrate that, by taking the singular limit, this phase-field based model can be reduced to a free boundary model, which is equivalent to the $L^2$-gradient flow model of self-propelled objects derived by the variational principle from the interfacial energy, which gives a physical interpretation to the phase-field model.

## Introduction

A self-propelled system is composed of a particle or droplet that exhibits self-propelled motion by consuming the free energy under non-equilibrium conditions. Such a particle or droplet can move by the internal mechanism; some can move by deforming themselves, and others move by changing the characters in the neighbouring field. Motions of living organisms, such as birds, insects, bacteria, and cells^1–4^ and those in non-living materials, such as camphor disks, swimming droplets, running droplets, and Janus particles^5–9^, are regarded as the motion in self-propelled systems. In recent years, various types of cell motility have been analysed through mathematical models, including keratocyte motility^10^, cell population motility^11–13^, and cell division^14^. Mathematical models for the motility of non-living materials are also investigated by constructing the mathematical model, for example, camphor particles^15,16^, pentanol droplets^17^, running oil droplets^18^, and blebbing oil droplets^19^. In this study, we focus on the spatio-temporal behaviours in self-propelled systems, such as the motion of camphor disks and pentanol droplets, where the mechanism for the self-propelled motion is believed to be primarily governed by surface tension gradient. Such surface-tension-driven systems are classified into two cases; the systems without shape deformation, like camphor disks, and those with shape deformation, like pentanol droplets. Formulations of two-dimensional motion models for the former case have already been made, and some comparisons of experimental results with those of mathematical models and mathematical analyses have been performed^20,21^.

On the other hand, as for mathematical modelling for the self-propelled system with shape deformation, the mathematical modelling approach has not yet been satisfactorily performed. A mathematical model based on the membrane motion model has been proposed for the droplet motion; however, it is not easy to handle as a model of droplet dynamics due to its substantial computational cost^22^. The other approach for the droplet motion is to adopt the lubrication approximation. Thiele and coauthors reported the two-dimensional model for the self-propelled droplet^23^, but no systematic study has not been demonstrated. Therefore, we aimed to construct a mathematical model suitable for numerical computation at a low cost that can be connected to the natural model from the physics viewpoint. For the purpose above, we adopted the Allen—Cahn equation^24^ to describe the droplet shape. By combining it with the equation for surfactant concentration used in previous studies, we constructed a mathematical model that can handle self-propelled object motion with shape change. The formulation of this mathematical model is a modelling method called the phase-field method, which has been used for crystal growth models in supercooled liquids^25^ and crystal interface motion models^26^. In recent years, there have been models^10,13,14^ to represent cell motility in living systems using the Allen–Cahn and the Cahn–Hilliard^27^ equations. They are also used in many other fields of materials science^28^. Although the phase-field model has been widely used and has the advantage that the cost for numerical computation is low, the model also has a disadvantage in that the correspondence between the actual physical quantity and the order parameter $\varphi$ remains unclear, which is because the order parameter is introduced artificially to connect the regions with different phases smoothly. Therefore, in this paper, we first construct the reaction–diffusion model for a self-propelled droplet using the volume-preserving Allen–Cahn type phase-field equation. Then, we derive the singular-limit model from the reaction–diffusion system model and confirm that the singular-limit model matches the self-propelled object motion model derived as the $L^2$-gradient flow based on the variational principle. From these results, we can demonstrate that the reaction–diffusion system model is an $\varepsilon$-approximation of the $L^2$-gradient flow model, which supports the physical meaning of the constructed mathematical model based on the Allen–Cahn type phase-field equation.

This study proposes the following mathematical model for a self-propelled object using the volume-preserving Allen–Cahn equation:

1 $$\begin{aligned} {\left\{ \begin{array}{ll} \varepsilon ^{2} \tau \frac{\partial \varphi }{\partial t} = \varepsilon ^2 \sigma ^2 \triangle \varphi + \varphi (1 - \varphi )(\varphi - a(S[\varphi ](t), u, \varepsilon )), &{} \varvec{x} \in \Omega ,\quad t>0, \\ \frac{\partial u}{\partial t} = d_u \triangle u - (k_1 + k_2) u + k_3 s_0\varphi , &{} {\varvec{x}} \in \Omega ,\quad t>0, \end{array}\right. } \end{aligned}$$

where

$$\begin{aligned} \triangle = \dfrac{\partial ^2 }{\partial x^2} + \dfrac{\partial ^2 }{\partial y^2}, \end{aligned}$$

and

2 $$\begin{aligned}{} & {} a(S[\varphi ](t), u, \varepsilon ) = \frac{1}{2} + \varepsilon \left( - \frac{1}{\gamma _0} \gamma (u) + S[\varphi ](t) \right) , \nonumber \\{} & {} S[\varphi ](t) = S_{\alpha }[\varphi ](t) =\frac{\alpha }{|\Omega |} \left( \int _{\Omega }\varphi (\varvec{x},t) d\varvec{x} - \int _{\Omega } \varphi (\varvec{x}, 0) d\varvec{x} \right) . \end{aligned}$$

Here, $\varphi (\varvec{x},t)$ represents the position and shape of the self-propelled droplet by defining $1/2\le \varphi (\varvec{x},t)\le 1$ as the inside of the droplet and $0\le \varphi (\varvec{x},t)<1/2$ as the water surface. $u(\varvec{x},t)$ represents the surface density of surfactant molecules on the water surface. $d_u$, $k_1$, $k_2$, and $k_3$ are the diffusion coefficient, dissolution rate, sublimation rate, and supply rate of surfactant molecules, respectively; $s_0$ is the density of self-propelled surfactant molecules inside the droplet; $\tau$, $\sigma$, and $\varepsilon$ are positive constants such that $0<\varepsilon \ll 1$. $\Omega$ is a bounded region in $\mathbb {R}^2$. The first equation for the time evolution of $\varphi$ represents the time evolution of the droplet shape. Since this equation is obtained based on the Allen–Cahn equation, the profile $\varphi$ should have the regions with $\varphi \simeq 0$ and $\varphi \simeq 1$ and the smoothly connecting boundary region, which correspond to the inside of the droplet, water surface and the periphery of the droplet, respectively. Function *a* in Eq. (2) represents the driving force by the surface tension at the object’s periphery and also keeps the object’s volume almost constant.

The equation for *a* is constructed so that greater $\gamma (u)$ and the smaller volume decrease *a*, which drives the droplet periphery outward. As for the time-evolution equation for *u*, the first term of the righthand side shows the diffusion of the surfactant molecules at the water surface. Since the surface tension gradient induces the Marangoni convection^29^, the transport of the surfactant molecules may be partly caused by the convection. However, this transport can be incorporated into the effective diffusion coefficient, which describes the overall surfactant molecule transformation^30^. The second term corresponds to the decrease in concentration by dissolution to the aqueous bulk phase and sublimation to the gas phase. We assume that the dissolution and sublimation occur proportionally to the surface concentration *u*. The last term shows the supply of surfactant molecules from the object. Here we assume that the supply is proportional to $\varphi$ since the supply only occurs in the region where the object covers. Such a quasi-conservative reaction–diffusion system was first proposed by Krischer et al.^31^. Previous results for the volume-preserving Allen–Cahn equation proved the existence and uniqueness of the classical solution and the boundedness of the solution^32^. Moreover, the mean curvature flow is derived using the singular limit^33^. Furthermore, the model (1) mentioned above, except the second equation, is also considered in terms of the geometric measure theory^34,35^, which shows that the singular limit becomes the volume-preserving mean curvature flow.

First, we present a mathematical model for a self-propelled object using the volume-preserving Allen–Cahn type phase-field equation and demonstrate by numerical simulations that the model can describe the motion of self-propelled objects with varying deformability. Then, we present a free boundary problem in which a closed curve represents the self-propelled object by performing a singular perturbation expansion. Numerical computations for this model show how the self-propelled motion can be reproduced. We also confirm numerically that the parameter $\sigma$ can control the interface change. Although free boundary problems obtained from the singular limit have been reported for reaction–diffusion systems with two variables^36–38^, ones with integral terms, such as Eq. (1), have not been known until now. We then define the interfacial energy of the water surface, the length energy of the self-propelled object, and the area-preserving energy. Based on the variational principle, we derive an $L^2$-gradient flow model representing the self-propelled object as a closed curve, showing that this mathematical model is consistent with the free boundary problem. We observe that the mathematical model using the volume-preserving Allen–Cahn equation approximates the $L^2$-gradient flow model, which corresponds to the free energy form. Finally, we summarise this study and discuss future work.

## Results

### Construction of reaction–diffusion model for the motion of a self-propelled object

In this section, we propose a phase-field type reaction–diffusion model that describes the motion of a self-propelled object. Here, the dimensionless model is shown, whereas the non-dimensionalisation from the dimensional model is shown in “Methods”. In our model, we set the order parameter $\varphi (\varvec{x},t)$, which defines $1/2\le \varphi (\varvec{x},t)\le 1$ as the self-propelled object and $0\le \varphi (\varvec{x},t)<1/2$ as the water surface. $u(\varvec{x},t)$ is the concentration of surfactant molecules supplied by the self-propelled object. Then, we obtain the following non-dimensionalised mathematical model from (1)–(2) (see “Methods”):

3 $$\begin{aligned} {\left\{ \begin{array}{ll} \varepsilon ^{2} \tau \frac{\partial \varphi }{\partial t} = \varepsilon ^2 \sigma ^2 \triangle \varphi + \varphi (1 - \varphi )(\varphi - a(S[\varphi ](t), u, \varepsilon )), &{} \varvec{x} \in \Omega ,\quad t>0, \\ \frac{\partial u}{\partial t} = \triangle u - k u + \varphi , &{} \varvec{x} \in \Omega ,\quad t>0, \end{array}\right. } \end{aligned}$$

where

4 $$\begin{aligned}&a(S[\varphi ](t), u, \varepsilon ) = \frac{1}{2} + \varepsilon \left( - \gamma (u) + S[\varphi ](t) \right) , \quad S[\varphi ](t) = S_{\alpha }[\varphi ](t) = \alpha \left( \int _{\Omega }\varphi (\varvec{x},t) d\varvec{x} - S_0\right) ,\nonumber \\&\gamma (u) = \frac{1}{1 + (u/u_1)^m} + \gamma _0, \quad S_0 = \int _{\Omega }\varphi _0(\varvec{x}) d\varvec{x} = \int _{\Omega }\varphi (\varvec{x}, 0) d\varvec{x}. \end{aligned}$$

The initial values for the mathematical model (3) are defined as follows:

5 $$\begin{aligned} \varphi (\varvec{x}, 0) = \varphi _0(\varvec{x}),\quad u(\varvec{x}, 0) = u_0(\varvec{x}),\quad \varvec{x}\in \overline{\Omega }:=\Omega \cup \partial \Omega , \end{aligned}$$

where $\partial \Omega$ represents the boundary of $\Omega$, $\varphi _0$ is a function with compact support, for example, given as

$$\begin{aligned} \varphi _0(\varvec{x}) = \frac{1}{2}\left( 1 + \tanh \left( \frac{r_0 - \Vert \varvec{x} - \varvec{x}_0 \Vert }{\delta } \right) \right) , \quad \varvec{x}_0\in \Omega , \quad 0<\delta \ll 1. \end{aligned}$$

### Numerical computation by the reaction–diffusion model

Numerical computations are performed on the reaction–diffusion model (3)–(4) under the initial condition (5) to investigate the properties of the mathematical model. Since the width of the interface of $\varphi$ is $O(\varepsilon \sigma )$, $\varepsilon$ and $\sigma$ are given so that $\varepsilon \sigma =0.025$.

**Figure 1:**
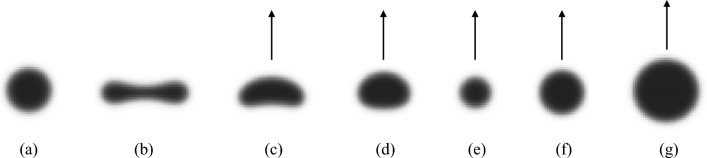
The typical profile of $\varphi$ for reaction–diffusion system (3) where parameters are $k = 10.0, u_1 = 0.05, \alpha = 1000.0$ and $m=2$ except $\sigma , \tau$ and $S_0$: (**a**) disk-shaped standing spot ($\sigma = 0.05, \tau =0.05, S_0 = 1.0$), (**b**) dumbbell-shaped standing spot ($\sigma = 0.05, \tau = 0.005, S_0 = 1.0$), (**c**) banana-shaped travelling spot ($\sigma = 0.05, \tau = 0.0008, S_0 = 1.0$), (**d**) rice-ball-shaped travelling spot ($\sigma = 0.05, \tau = 0.0001, S_0 = 1.0$), (**e**) almost disk-shaped travelling spot ($\sigma = 0.05, \tau = 0.003, S_0 = 0.5$), (**f**) almost disk-shaped travelling spot ($\sigma = 1.0, \tau = 0.01, S_0 = 1.0$), (**g**) almost disk-shaped travelling spot with large velocity ($\sigma = 1.0,\tau = 0.0025, S_0 = 2.0$) (see supplement movies).

First, consider the case $\sigma =0.05$ (i.e., $\varepsilon =0.5$) with $\tau$ and the integral $S_0$ of the initial function as free parameters. When $S_0=1$, a disk-shaped standing spot appears stably, as shown in Fig. 1a for large $\tau$. As $\tau$ gradually decreases, the disk-shaped standing spot becomes unstable, and a dumbbell-shaped standing spot appears, as shown in Fig. 1b. As $\tau$ is further decreased, a banana-shaped travelling spot solution appears (Fig. 1c). Then, as $\tau$ is decreased, the velocity increases, and the banana-shaped travelling spot deforms into a rice-ball-shaped travelling spot (Fig. 1d). When $S_0=0.5$, as $\tau$ gradually decreases, a travelling spot appears, which is almost disk-shaped, from the disk-shaped standing spot (Fig. 1e). The above result shows that a travelling spot bifurcates from the disk-shaped standing spot supercritically.

As $\tau$ is further decreased, a faster rice-ball-shaped travelling spot appears. Next, we consider the case $\sigma =1$ (i.e., $\varepsilon =0.025$). For $S_0=1$, a disk-shaped standing spot appears for large $\tau$, but as $\tau$ is gradually decreased, the disk-shaped standing spot becomes unstable, and a travelling spot close to the disk appears (Fig. 1f). Furthermore, even when $S_0=2$, although the travelling velocity is high, the deformation is slightly elliptical, but the convexity is maintained (Fig. 1g). To confirm that the disk shape is maintained for larger values of $\sigma$, we set $\sigma =5$ (i.e., $\varepsilon =0.005$), and indeed, a travelling spot close to a disk appears for small values of $\tau$. The results show that an almost disk-shaped travelling spot emerges even at small $\tau$. Furthermore, these spot solutions are found to asymptote to a constant velocity from a suitable compact support function $\varphi _0(\varvec{x})$, as shown in Supplemental Fig. 1a–g.

**Figure 2:**
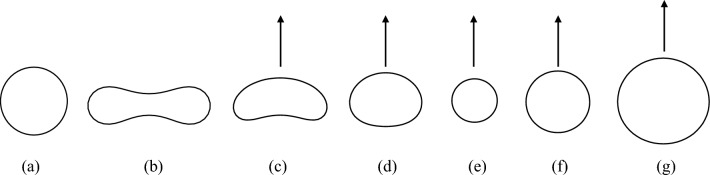
The profile of typical solutions for (6) where parameters are $k = 10.0, u_1 = 0.05, \alpha = 1000.0$ and $m=2$ except $\sigma$, $\tau$, and $S_0$: (**a**) radial symmetric standing spot ($\sigma =0.05$, $\tau =0.05, S_0 = 1.0$), (**b**) dumbbell-shaped standing spot ($\sigma =0.05$, $\tau = 0.01, S_0 = 1.0$), (**c**) banana-shaped travelling spot ($\sigma =0.05$, $\tau = 0.001, S_0 = 1.0$), (**d**) rice-ball-shaped travelling spot ($\sigma =0.05$, $\tau = 0.0001, S_0 = 1.0$), (**e**) near-circular travelling spot($\sigma =0.05$, $\tau = 0.0025, S_0 = 0.5$), (**f**) near-circular travelling spot ($\sigma =1.0$, $\tau =0.01$, $S_0=1.0$), (**g**) near-circular travelling spot ($\sigma =1.0$, $\tau =0.0025$, $S_0=2.0$) (see supplementary movies).

The banana-shaped travelling spot, as shown in Fig. 1c, is similar to a pentanol droplet exhibiting the translational motion (Fig. 1(c-1) in the Reference^17^, Fig. 1 in the Reference ^39^); the elliptic travelling spot, as shown in Fig. 1d, appears in the translational motion of a relatively small pentanol droplet (Fig. 2a in the Reference^22^); and the travelling spot close to a disk, as shown in Fig. 1f, appears in the translational motion of an ethyl salicylate droplet (Fig. 1 in the Reference^40)^. Moreover, the travelling spot near the disk shape with high velocity, as shown in Fig. 1g, approximates the non-deformable self-propelled objects such as solid camphor disks. These results suggest that the mathematical model (3)–(4) can describe a wide range of motions, from deformable self-propelled objects such as droplets to non-deformable self-propelled objects such as solid camphor disks, using $\sigma$ as a parameter.

### Reduction to the singular-limit model

In this section, we clarify that the shape of the spot pattern in (3) can be controlled by using only the parameter $\sigma$. To this end, we derive a singular limit model as $\varepsilon$ tends to 0 in (3) and perform numerical simulations for the singular limit model.

The first equation of  (3) is equivalent to the “Allen–Cahn equation”, provided that $a(S[\varphi ](t), u, \varepsilon )\equiv 1/2$. It is well-known that, as $\varepsilon$ tends to 0 under this setting, the motion of the level set at $\varphi (\varvec{x},t)=1/2$ is close to that of the mean curvature flow. This section investigates how the solution of (3) and the level set at $\varphi (\varvec{x},t)=1/2$ behave as $\varepsilon$ tends to 0. Let $\Gamma (t)$ be a Jordan curve depending on the time *t* in $\mathbb {R}^2$, that is,

$$\begin{aligned} \Gamma (t):= \{\varvec{x} \in \Omega \ | \ \varphi (\varvec{x},t) = 1/2\}, \end{aligned}$$

and $\Omega ^{\textrm{in}}(t)$ be a bounded domain with a smooth boundary $\Gamma (t)$. Then, the singular limit of (3) leads to the following free boundary problem, composed of the interface equation and the reaction–diffusion equation:

6 $$\begin{aligned} {\left\{ \begin{array}{ll} V = -\sigma ^2 \dfrac{\kappa }{\tau } + \dfrac{\sigma \sqrt{2}}{\tau } \left( \gamma (u) + \alpha \left( { S_0} - |\Omega ^{\textrm{in}}(t)| \right) \right) , &{} \varvec{x}\in \Gamma (t),\quad t>0, \\ \dfrac{\partial u}{\partial t} = \triangle u - k u + F(\varvec{x}, \Omega ^{\textrm{in}}(t) ), &{} \varvec{x}\in \Omega \setminus \Gamma (t),\quad t>0, \end{array}\right. } \end{aligned}$$

where $$S_0 = |\Omega ^{\textrm{in}}(0)|$$ and

7 $$\begin{aligned} F(\varvec{x}, \Omega ^{\textrm{in}}(t)) = {\left\{ \begin{array}{ll} 1, &{} \varvec{x}\in \Omega ^{\textrm{in}}(t), \\ 0, &{} \varvec{x}\not \in \Omega ^{\textrm{in}}(t). \end{array}\right. } \end{aligned}$$

The derivation of (6) is shown in “Methods”.

### Numerical computation by the singular limit model

In this section, we perform simulations of the singular limit Eq. (6) and show that the parameter $\sigma$ controls the shape of travelling spots that appear in (6).

To begin with, let $\sigma = 0.05$. We here regard $\tau$ and $S_0$ defined by the initial function as free parameters. When $S_0 = 1$, if $\tau$ is sufficiently large, the disk-shaped stationary solution, which is stable, appears (Fig. 2a). As $\tau$ gradually decreases, as in the reaction–diffusion model, a dumbbell-shaped standing spot solution first appears (see Fig. 2b), followed by a banana-shaped travelling spot (see Fig. 2c). As $\tau$ is further reduced, a rice-ball-shaped travelling spot eventually appears (see Fig. 2d). On the other hand, as $S_0=0.5$, the situation is slightly different. When $\tau$ decreases gradually, the travelling spot appears as well as $S_0 = 1$, but the banana-shaped one does not appear. Only travelling spots, whose shapes are almost disk-shaped, are observed (Fig. 2e). Next, let $\sigma = 1.0$. Under the condition of $S_0=1$, as $\tau$ decreases gradually, the almost disk-shaped travelling spot appears (Fig. 2f). As $S_0 = 2$, the shape of the spot changes from a disk to an ellipse and preserves the convexity (Fig. 2g). Similar to the model (3)–(4), the singular limit equation (6) also asymptotes to a constant velocity and a constant shape spot from suitable initial values, as shown in Supplemental Figs.  2a–g and  3I,II.

### Formulation of self-propelled motion model by $L^2$-gradient approach

In this section, we construct a gradient flow model of a self-propelled motion to give a physical meaning to our proposed model (3). Let $L(\partial \Omega ^{\textrm{in}})$, $E(\Omega ^{\textrm{in}})$ and $S(\Omega ^{\textrm{in}})$ be the surface energy of water surface, the perimeter energy of self-propelled particles and the energy of area-preserving, respectively, which are given by

8 $$\begin{aligned} L(\partial \Omega ^{\textrm{in}}) = \int _{\partial \Omega ^{\textrm{in}}} ds, \quad E(\Omega ^{\textrm{in}}) = \int _{\Omega \backslash \Omega ^{\textrm{in}}} \gamma (u(\varvec{x},t)) d\varvec{x}, \quad S(\Omega ^{\textrm{in}}) = \beta (|\Omega ^{\textrm{in}}| - |\Omega ^{0}|)^2, \end{aligned}$$

where $s=s(\varvec{x},t)$ stands for the arc-length of $\varvec{x} \in \partial \Omega ^{\textrm{in}}$, $\partial \Omega ^{\textrm{in}}= \partial \Omega ^{\textrm{in}}(t)$, $\Omega ^{\textrm{in}}=\Omega ^{\textrm{in}}(t)$ and

$$\begin{aligned} |\Omega ^{\textrm{in}}| = \int _{\Omega ^{\textrm{in}}(t)} d\varvec{x}, \quad |\Omega ^{0}| = \int _{\Omega ^{\textrm{in}}(0)} d\varvec{x}. \end{aligned}$$

Define the total energy $H(\bar{\Omega }^{\textrm{in}})$ by

9 $$\begin{aligned} H(\bar{\Omega }^{\textrm{in}}): = L(\partial \Omega ^{\textrm{in}}) + E(\Omega ^{\textrm{in}}) + S(\Omega ^{\textrm{in}}). \end{aligned}$$

Since we obtain from (19) (see “Methods”) that

$$\begin{aligned} \frac{\delta E}{\delta p(s)} = - \gamma (s, t), \quad \frac{\delta L}{\delta p(s)} = \kappa (s, t), \quad \frac{\delta S}{\delta p(s)} = 2\beta \left( |\Omega ^{\textrm{in}}| - |\Omega ^{0}| \right) , \end{aligned}$$

assuming that the energy of $\partial \Omega ^{\textrm{in}}(t)$ decreases in time, we conclude that

10 $$\begin{aligned} V(s, t) = - K \left( \kappa (s, t) - \gamma (s, t) + 2\beta \left( |\Omega ^{\textrm{in}}| - |\Omega ^{0}| \right) \right) . \end{aligned}$$

Combining the interface motion (10) with the reaction–diffusion model for surfactants and choosing

$$\begin{aligned} K = \frac{\sigma ^2}{\tau },\quad \gamma (s, t) = \frac{\sqrt{2}}{\sigma }\gamma (u(s, t)), \quad \beta = \frac{\alpha }{\sqrt{2} \sigma },\quad { |\Omega ^{0}| = S_0}, \end{aligned}$$

we obtain the following $L^2$-gradient flow model:

11 $$\begin{aligned} {\left\{ \begin{array}{ll} V = - \sigma ^2 \dfrac{\kappa }{\tau } + \dfrac{\sqrt{2} \sigma }{\tau } \left( \gamma (u) + \alpha ( {S_0 } - |\Omega ^{\textrm{in}}(t)| ) \right) , &{} \varvec{x}\in \Gamma (t),\quad t>0, \\ \dfrac{\partial u}{\partial t} = \triangle u - k u + F(x, \Omega ^{\textrm{in}}(t) ), &{} \varvec{x}\in \Omega \setminus \Gamma (t),\quad t>0, \end{array}\right. } \end{aligned}$$

which is the same as (6). The detailed calculation is shown in “Methods”. This result shows that our proposed model (3) is an $\varepsilon$-approximate model of the gradient flow derived from the energy variation. The equation of motion of the interface is derived from the variational principle, where time evolution occurs in the direction of decreasing total energy. However, the variational principle is not applied to the concentration field. Note, therefore, the energy of the entire system is not conserved. Furthermore, the system is not stationary at the energy minimum.

## Conclusion

In this study, we have demonstrated that a phase-field based reaction–diffusion model of self-propelled motion can be considered as an approximation of an energy-based gradient flow model. We have shown that a free boundary model (6) can be derived as a singular limit from the reaction–diffusion model (3) and that this derived model is exactly the same as the model (11) derived from the variational principle applied to the interfacial energy of the self-propelled object. This result gives a phase-field based model, typically regarded as a phenomenological model, a physical interpretation tied to the interfacial energy. Furthermore, we have demonstrated that, by adjusting a single control parameter $\sigma$, our reaction–diffusion model yields both deformed and near-circular traveling spots, which means that our model can represent both deformable droplet motion and solid camphor disk motion. Thus, our work offers a more comprehensive and theoretically grounded framework for the modeling of self-propelled objects.

In our model, the bifurcation from a standing to travelling spot occurs when the parameter $\tau$ is decreased. Intuitively, if a self-propelled object has a circular shape, the surface tension should be balanced due to the symmetric diffusion of surfactant from the object. However, when the object moves slightly in a certain direction, the concentration field behind it increases, and the surface tension behind it decreases, causing an imbalance of forces. When the imbalance overwhelms the effect of diffusion attempting to restore symmetry, it continues to move (see the Supplementary movies). For deformable objects such as droplets, the motion can also break the symmetry of the shape; for solid objects, like camphor particles, only the concentration field breaks the symmetry. The supplementary movies shows that, even if the self-propelled object maintains a symmetrical shape, the motion continues as the concentration field profile becomes asymmetrical. This illustrates how our model can exhibit both deformed and circular travelling spots.

Our current model cannot handle arbitrarily shaped solid objects such as an elliptic-shaped camphor particle, which requires that the steady state of the phase-field model be controlled in a desired way. Whether it is possible to represent self-propelled object motions that maintain elliptical or other shapes within our framework would be an interesting future work. We would then like to compare the results of the previous analysis^20,21^ of elliptical camphor motion with the current model.

Moreover, the stable dumbbell-shaped standing spot bifurcated from the stable disk-shaped standing spot has been found by numerical calculation in the self-propelled motion of reaction–diffusion systems. Figure 1b shows that the stable dumbbell-shaped standing spot has been reported for the reaction–diffusion equation with the spatially inhomogeneous nonlinear term^41^. However, only the unstable dumbbell-shaped standing spot has been reported for spatially homogeneous reaction–diffusion systems^42,43^. In the future, we will analyse the existence and stability of stationary solutions to the reaction–diffusion type self-propelled object motion model (3)–(4) and the free boundary model (6) and investigate the dynamics of the mathematical model in detail by analysing the bifurcation structure to the travelling spot. In the case of splitting phenomena such as observed in droplets, the volume-preserving reaction–diffusion model (3)–(4) can be used to confirm the splitting of self-propelled objects numerically. However, this model does not conserve the volume of individual self-propelled objects. To describe the motion of multiple self-propelled objects after splitting, it is necessary to extend the mathematical model to handle the phenomenon of splitting, for example, by preparing a volume-preserving model for the number of self-propelled objects as a simple extension.

## Methods

### Non-dimensionalisation of the reaction–diffusion model with dimension

We perform the following non-dimensionalisation on the reaction–diffusion model (1):

$$t' = k_3 t, \quad \varvec{y} = \sqrt{\frac{k_3}{d_u}} \varvec{x}, \quad U(\varvec{y}, t') = \frac{u(\varvec{x},t)}{s_0},\quad \phi (\varvec{y}, t') = \varphi (\varvec{x}, t),\quad \Omega '= \left\{ \varvec{y} \ |\ \varvec{y} = \sqrt{k_3/d_u} \varvec{x},\ \varvec{x} \in \Omega \right\} .$$

Then, the model Eqs. (1) and (2) become

$$\begin{aligned} {\left\{ \begin{array}{ll} \varepsilon ^{2} \tau ' \dfrac{\partial \phi }{\partial t'} = \varepsilon ^2 (\sigma ')^2 \triangle _{\varvec{y}} \phi + \phi (1 - \phi )(\phi - a(S[\varphi ](t'), U, \varepsilon )), &{} \varvec{y} \in \Omega ',\quad t'> 0, \\ \dfrac{\partial U}{\partial t' } = \triangle _{\varvec{y}} U - k' U + \phi , &{} \varvec{y} \in \Omega ',\quad t' > 0, \end{array}\right. } \end{aligned}$$

and

$$\begin{aligned} a(S[\varphi ](t'), U, \varepsilon )&= \frac{1}{2} + \varepsilon \left( - \gamma (U) + S[\varphi ](t') \right) ,\\ S[\varphi ](t')&= S_{\alpha }[\varphi ](t') = \alpha ' \left( \int _{\Omega '}\phi (\varvec{y},t') d\varvec{y} - \int _{\Omega '} \phi (\varvec{y}, 0) d\varvec{y} \right) , \\ \gamma (U)&= \frac{1}{1 + ( U/u_1' )^m} + \Gamma _0 \end{aligned}$$

respectively, where

$$\begin{aligned} \tau ' = k_3\tau ,\quad \sigma ' = \sqrt{\frac{k_3}{d_u}}\sigma , \quad \alpha ' = \frac{d_u}{k_3}\frac{\alpha }{|\Omega |}, \quad k' = \frac{k_1 + k_2}{k_3}, \quad u_1' = \frac{u_1}{s_0}, \quad \Gamma _0 = \frac{\gamma _0}{\gamma _1}. \end{aligned}$$

We obtain the following by re-writing the non-dimensionalized mathematical model with the original variables:

$$\begin{aligned} {\left\{ \begin{array}{ll} \varepsilon ^{2} \tau \dfrac{\partial \varphi }{\partial t} = \varepsilon ^2 \sigma ^2 \triangle \varphi + \varphi (1 - \varphi )(\varphi - a(S[\varphi ](t), u, \varepsilon )), &{} \varvec{x} \in \Omega ,\quad t>0, \\ \dfrac{\partial u}{\partial t} = \triangle u - k u + \varphi , &{} \varvec{x} \in \Omega ,\quad t>0, \end{array}\right. } \end{aligned}$$

where

$$\begin{aligned} a(S[\varphi ](t), u, \varepsilon )&= \frac{1}{2} + \varepsilon \left( - \gamma (u) + S[\varphi ](t)) \right) , \\ S[\varphi ](t)&= S_{\alpha }[\varphi ](t) = \alpha \left( \int _{\Omega }\varphi (\varvec{x},t) d\varvec{x} - \int _{\Omega } \varphi (\varvec{x}, 0) d\varvec{x} \right) , \\ \gamma (u)&= \frac{1}{1 + (u/u_1)^m} + \gamma _0. \end{aligned}$$

### Method for the numerical calculation by the reaction–diffusion model

The region $\Omega _{}$ is fixed as $\Omega =(-L_x,L_x)\times (-L_y,L_y)$, $L_x=5$, $L_y=2$, and the periodic boundary condition is imposed. The parameters $\alpha$ and $u_1$ are set to $\alpha =1000$, $u_1=0.005$ for all settings. Numerical computations of the reaction–diffusion model (3)–(4) were performed using the alternating direction implicit method^44^.

### Formal derivation of the singular-limit model

In what follows, we demonstrate the interface equation by the formal argument.

#### Outer expansion

Assume that $\varphi$ and *u* have the following expansions away from the interface $\Gamma (t)$ :

12 $$\begin{aligned} \varphi =\varphi ^0+\varepsilon \varphi ^1+O(\varepsilon ^2), \quad u=u^0+\varepsilon u^1+O(\varepsilon ^2). \end{aligned}$$

Substituting the expansion (12) into (3) and collecting the term $\varepsilon ^{0}$, we know that $\varphi ^0$ satisfies $\varphi ^0(1-\varphi ^0)(\varphi ^0 - 1/2)=0$, and hence $\varphi ^0=1$ in $\Omega ^{\textrm{in}}(t)$ and $\varphi ^0=0$ in $\Omega \setminus \Omega ^{\textrm{in}}(t)$. Similarly, $u^0$ satisfies

13 $$\begin{aligned} \displaystyle \frac{\partial u^0}{\partial t} = \triangle u^0 - k u^0 + \varphi ^0, \quad \varvec{x} \in \Omega \setminus \Gamma (t), \quad t>0. \end{aligned}$$

#### Inner expansion

We consider the formal expansion near $\Gamma (t)$. Let *p* be an arc length parameter of $\Gamma (t)$ (counter-clockwise) and *q* be a distance parameter along the outward normal direction at the point $\varvec{x}_0(p) \in \Gamma (t)$. Any point $\varvec{x}$ in the neighborhood of $\Gamma (t)$ is represented by $\varvec{x}=\varvec{x}(p,q)=\varvec{x}_0(p)+q \varvec{\nu }(p)$, where $\varvec{\nu }(p)$ is the outer normal unit vector at $\varvec{x}_0(p)$. From this, two inverse functions $p=P(\varvec{x},t)$ and $q=Q(\varvec{x},t)$ for $\varvec{x} \in \Gamma (t)$ are defined. Denote $\Phi$ and *U* by

$$\begin{aligned}&\Phi (p,q,t) : =\Phi (P(x,y,t), Q(x,y,t)/\varepsilon , t)=\varphi (\varvec{x},t),\\&U(p,q,t) : =U(P(x,y,t), Q(x,y,t)/\varepsilon , t)=u(\varvec{x},t) \end{aligned}$$

Substituting these two functions into (3), we have

$$\begin{aligned} \displaystyle \varepsilon ^2 \tau \left( \Phi _p P_{t} + \Phi _q \frac{Q_{t}}{\varepsilon } + \Phi _{t} \right)&= \varepsilon ^2 \sigma ^2 \left( \Phi _{pp}|\nabla P |^2 + \Phi _p \triangle P + \frac{1}{\varepsilon ^2}\Phi _{qq} + \frac{1}{\varepsilon }\Phi _q \triangle _p Q \right) \\&\quad + \Phi (1 - \Phi )(\Phi - a(S[\varphi ](t), U, \varepsilon )). \end{aligned}$$

Here $\triangle _p$ stands for Laplace–Beltrami operator. We collect the $\varepsilon ^1$ term and obtain the relation

14 $$\begin{aligned} \displaystyle \sigma ^2\Phi _{qq} + f(\Phi )+\varepsilon (\sigma ^2 \kappa \Phi _q + \tau V \Phi _q + \Phi (1-\Phi )( \gamma (U)-S[\varphi ](t))) +O(\varepsilon ^2) = 0, \end{aligned}$$

where $f(\Phi )=\Phi (1 - \Phi )(\Phi -1/2)$, $\kappa$ is the mean-curvature on $\Gamma (t)$, *V* is the normal velocity of $\Gamma (t)$. Assume that $\Phi$ and *U* have the following expansions near the interface $\Gamma (t)$ :

15 $$\begin{aligned} \Phi =\Phi ^0+\varepsilon \Phi ^1+O(\varepsilon ^2), \quad U=U^0+\varepsilon U^1+O(\varepsilon ^2). \end{aligned}$$

Substituting (15) into (14), we know that $\Phi ^0$ and $\Phi ^1$ satisfy

16 $$\begin{aligned}&\sigma ^2 \Phi _{qq}^0 + f(\Phi ^0) = 0, \end{aligned}$$

17 $$(\sigma ^2 \kappa + \tau V )\Phi _q^0 + \sigma ^2 \Phi _{qq}^1 + f'(\Phi ^0)\Phi ^1 + \Phi ^0(1-\Phi ^0)(\gamma (U^0)-S[\varphi ^0](t)) = 0,$$

where $S[\varphi ^0](t)=\alpha ( \int _{\Omega }\varphi ^0(\varvec{x},t) \,d\varvec{x} -\int _{\Omega }\varphi ^0(\varvec{x},0) \,d\varvec{x}$). Meanwhile, by repeating the same process for *U*, we obtain that

$$\begin{aligned} \varepsilon ^{-2}d_uU^0_{qq}+\varepsilon ^{-1}Q_tU^0_{q} + O(1)=0 \end{aligned}$$

By the matched conditions of the inner and outer solutions, *U* and $\Phi$ need to satisfy some boundary conditions. Then it holds $U^0(p,q,t) \equiv u^0(\varvec{x}(p,0),t)$, and then $U^0$ is independent of *q*. Moreover, since the function $\Phi ^0$ satisfies (16) and the matched conditions, it holds that $\lim _{q \rightarrow -\infty }\Phi ^0=0$, $\lim _{q \rightarrow +\infty }\Phi ^0=1$ and $\lim _{q \rightarrow \pm \infty }U^0=\lim _{q\rightarrow \pm 0 }u^0(\varvec{x}(p,q),t)$ and hence (17) is rewritten by

$$\begin{aligned} \displaystyle \sigma ^2 \Phi _{qq}^1 + f'(\Phi ^0)\Phi ^1 = (- (\sigma ^2 \kappa + \tau V ) + \sqrt{2}\sigma (\gamma (U^0) - S[\varphi ^0](t)))\Phi _q^0. \end{aligned}$$

Differentiating both sides of (16) in *q*, we have $\sigma ^2 (\Phi _{q}^0)_{qq} + f'(\Phi ^0)\Phi ^0_q = 0$. Set $\displaystyle L = \sigma ^2 d^2/dq^2 + f'(\Phi ^0)$, and then *L* is a self-adjoint operator (see Lemma 2.2 in the reference^36^). Hence

$$\begin{aligned} 0&=(L\Phi ^0_q, \Phi ^1) =(\Phi ^0_q, L \Phi ^1) =(- (\sigma ^2 \kappa + \tau V ) + \sqrt{2}\sigma (\gamma (U^0) - S[\varphi ^0](t))) \Vert \Phi ^0_q \Vert _{L^2(\mathbb {R}^2)}^2 \end{aligned}$$

and the following interface equation is obtained :

18 $$\begin{aligned} \tau V = - \sigma ^2 \kappa + \sqrt{2}\sigma (\gamma (U^0) - S[\varphi ^0](t)). \end{aligned}$$

Combining (13) and (18), we obtain the free boundary problem (6).

### Method for the numerical computation by the singular-limit equation

The computational area is a rectangular area $\Omega =(-L_x, L_x)\times (-L_y, L_y)$, the boundary condition is periodic and the parameters $L_x, L_y, \alpha$ and $u_0$ are the same as in Fig. 1. The boundaries are approximated by polygonal curves in the numerical computations of the interface model (6). See the supplementary information for these details.

### Details in the formulation of self-propelled motion model by $L^2$ gradient approach

In what follows, we construct the gradient flow model (11) of a self-propelled motion. Recall that the definition of $L(\partial \Omega ^{\textrm{in}})$,$E(\Omega ^{\textrm{in}})$, $S(\Omega ^{\textrm{in}})$ and $H(\bar{\Omega }^{\textrm{in}})$ are given by (8) and (9). Assume that a small perturbation of $\partial \Omega ^{\textrm{in}}$ is given by

$$\begin{aligned} \partial \Omega ^{\textrm{in}}_{\varepsilon } = \varvec{y}(s) + \varepsilon p(s) \varvec{N}(s), \end{aligned}$$

where *s* is the arc length of $\partial \Omega ^{\textrm{in}}$, $\varepsilon$ is a small positive constant, *p*(*s*) is the distance function and ${\varvec{N}}(s)$ is the normal unit vector. Each first variation of (8) is represented by

19 $$\begin{aligned} \delta E(\Omega ^{\textrm{in}})&= - \int _{\partial \Omega ^{\textrm{in}}} \gamma (s, t) p(s, t) ds, \quad \delta L(\Omega ^{\textrm{in}}) = \int _{\partial \Omega ^{\textrm{in}}}\kappa (s, t) p(s, t) ds, \nonumber \\ \delta S(\Omega ^{\textrm{in}})&= \int _{\partial \Omega ^{\textrm{in}}} 2\beta \left( |\Omega ^{\textrm{in}}| - |\Omega ^{0}| \right) p(s) ds , \end{aligned}$$

respectively, where $\kappa$ is the curvature of $\partial \Omega ^{\textrm{in}}$. For more details, see the supplementary information. Here we remark that, in general, $\delta H(\bar{\Omega }^{\textrm{in}})$ satisfies

$$\begin{aligned} \delta H(\bar{\Omega }^{\textrm{in}}) = \int \frac{\delta H}{\delta u(x)} u(x) dx \end{aligned}$$

Using *p*(*x*) instead of *u*(*x*), we have

$$\begin{aligned} \delta H(\bar{\Omega }^{\textrm{in}}) = \delta L(\Omega ^{\textrm{in}}) + \delta E(\Omega ^{\textrm{in}}) + \delta S(\Omega ^{\textrm{in}}) = \int _{\partial \Omega ^{\textrm{in}}} \left( \frac{\delta L}{\delta p(s)} + \frac{\delta E}{\delta p(s)} + \frac{\delta S}{\delta p(s)}\right) p(s) ds . \end{aligned}$$

It follows from (19) that

$$\begin{aligned} \frac{\delta E}{\delta p(s)} = - \gamma (s, t), \quad \frac{\delta L}{\delta p(s)} = \kappa (s, t), \quad \frac{\delta S}{\delta p(s)} = 2\beta \left( |\Omega ^{\textrm{in}}| - |\Omega ^{0}| \right) . \end{aligned}$$

Assume that the energy of $\partial \Omega ^{\textrm{in}}(t)$ decreases in time, and then

$$\begin{aligned} \frac{\partial }{\partial t}p(s, t)&= - K \left( \frac{\delta L}{\delta p(s)} + \frac{\delta E}{\delta p(s)} + \frac{\delta S}{\delta p(s)}\right) = - K \left( \kappa (s, t) - \gamma (s, t) + 2\beta \left( |\Omega ^{\textrm{in}}(t)| - |\Omega ^{0}| \right) \right) . \end{aligned}$$

Since $\displaystyle \frac{\partial }{\partial t}p(s, t)$ is the normal velocity of $\partial \Omega ^{\textrm{in}}(t)$, we conclude that

20 $$\begin{aligned} V(s, t) = - K \left( \kappa (s, t) - \gamma (s, t) + 2\beta \left( |\Omega ^{\textrm{in}}(t)| - |\Omega ^{0}| \right) \right) . \end{aligned}$$

Combining the interface motion (20) with the dynamics model for surfactants, we obtain the following self-propelled motion model:

21 $$\begin{aligned} {\left\{ \begin{array}{ll} V = K( -\kappa + \gamma ) + 2K \beta ( |\Omega ^{0}| - |\Omega ^{\textrm{in}}(t)| ), &{} \varvec{x}\in \Gamma (t),\quad t>0, \\ \dfrac{\partial u}{\partial t} = \triangle u - k u + F(\varvec{x}, \Omega ^{\textrm{in}}(t) ), &{} \varvec{x}\in \Omega \setminus \Gamma (t),\quad t>0, \end{array}\right. } \end{aligned}$$

where $F(\varvec{x}, \Omega ^{\textrm{in}}(t))$ is given by (7). By choosing

$$\begin{aligned} K = \frac{\sigma ^2}{\tau },\quad \gamma (s, t) = \frac{\sqrt{2}}{\sigma }\gamma (u(s, t)), \quad \beta = \frac{\alpha }{\sqrt{2} \sigma }, \quad { |\Omega ^{0}| = S_0}, \end{aligned}$$

(21) is the same as (6). For the computation of the first variations of the energies $L(\partial \Omega ^{\textrm{in}})$, $E(\Omega ^{\textrm{in}})$, and $S(\Omega ^{\textrm{in}})$, see the supplementary information.

### Supplementary Information

Supplementary Figures.

Supplementary Video.

## Data Availability

The datasets used and/or analysed during the current study available from the corresponding author on reasonable request.
