# Supplementary material for: On the reaction–diffusion type modelling of the self-propelled object motion
Source: Sci Rep. 2023 Aug 3;13:12633. doi: 10.1038/s41598-023-39395-w (PMC10400585; doi:10.1038/s41598-023-39395-w)
Supplement: Supplementary file 1 — Supplementary Figures. [file 41598_2023_39395_MOESM1_ESM.pdf]

Supplementary Information

On the reaction-diffusion type modelling of the self-propelled object motion

Masaharu Nagayama, Harunori Monobe, Koya Sakakibara, Ken-Ichi Nakamura, Yasuaki Kobayashi, and Hiroyuki Kitahata

Suuplemental figures

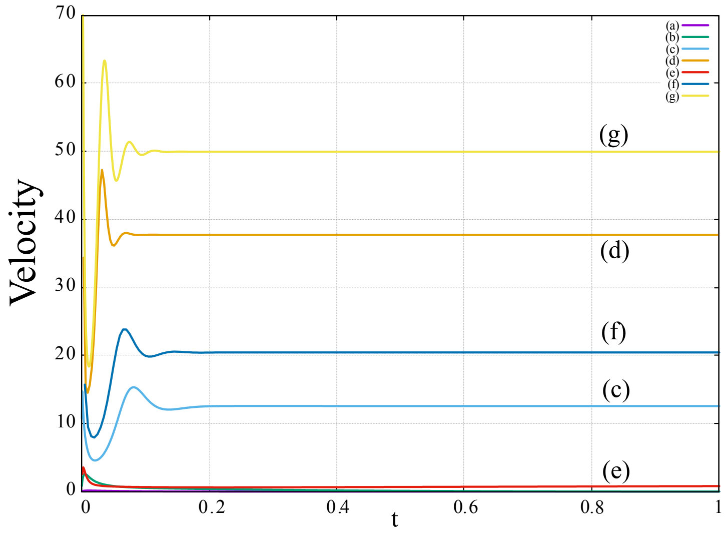

Supplementary Figure 1. Time evolution results of the velocity of the spot for Fig. 1(a)-(g). The parameters in (a)-(g) are the same as in Fig. 1(a)-(g).

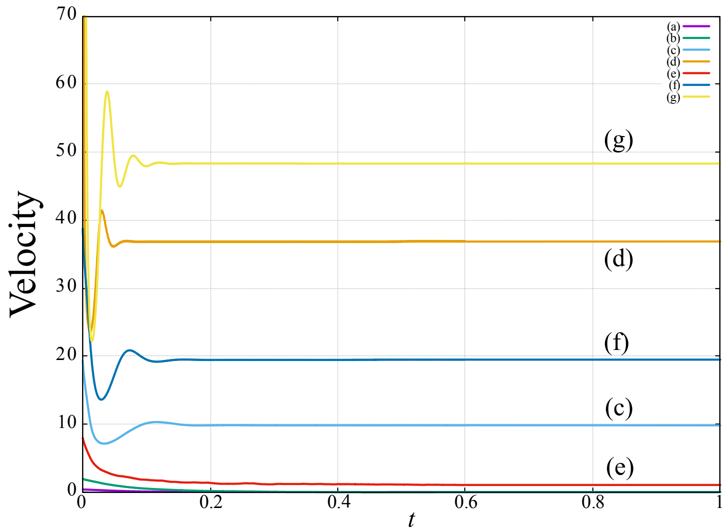

Supplementary Figure 2. Time evolution results of the velocity of the spot for Fig.2 (a)-(g). The parameters in (a)-(g) are the same as in Fig. 2(a)-(g).

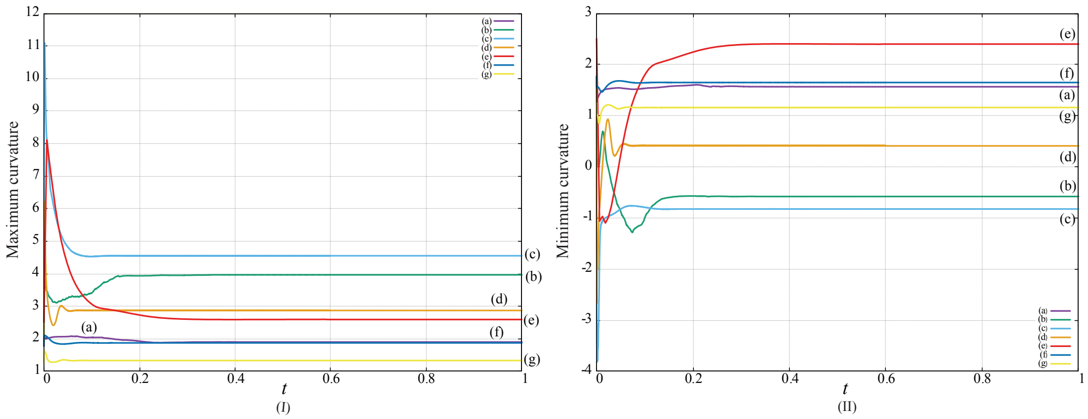

Supplementary Figure 3. Time evolution results of the maximum points (I) and minimum points(II) of curvature of spots for Fig. 2(a)-(g). The parameters in (a)-(g) are the same as in Fig. 2(a)-(g).

First variations of energies

In this section, we compute the first variations of the length, interfacial, and conservation energy.

The first variation of the length

Let Γ be a smooth closed curve in $\mathbb{R}^2$ and denote its parameterisation by $\boldsymbol{\gamma}\colon \mathbb{R}/\mathbb{Z} \to \mathbb{R}^2$. The length $L(\Gamma)$ of Γ is defined by

$$L(\Gamma) = L(\boldsymbol{\gamma}) := \int_0^1 |\partial_u \boldsymbol{\gamma}(u)|\, \mathrm{d}u.$$

Let us compute the L^2 variation of the length $L(\Gamma)$. To this end, take any smooth closed curve $\boldsymbol{\zeta}$. Then, we have

$$\begin{aligned}
\frac{\mathrm{d}}{\mathrm{d}\varepsilon} L(\boldsymbol{\gamma}+\varepsilon\boldsymbol{\zeta})\Big|_{\varepsilon=0} &= \frac{\mathrm{d}}{\mathrm{d}\varepsilon} \int_0^1 |\partial_u \boldsymbol{\gamma}(u) + \varepsilon \partial_u \boldsymbol{\zeta}(u)|\, \mathrm{d}u \Big|_{\varepsilon=0} \\
&= \int_0^1 \frac{(\partial_u \boldsymbol{\gamma}(u) + \varepsilon \partial_u \boldsymbol{\zeta}(u)) \cdot \partial_u \boldsymbol{\zeta}(u)}{|\partial_u \boldsymbol{\gamma}(u) + \varepsilon \partial_u \boldsymbol{\zeta}(u)|}\, \mathrm{d}u \Big|_{\varepsilon=0} \\
&= \int_0^1 \frac{\partial_u \boldsymbol{\gamma}(u) \cdot \partial_u \boldsymbol{\zeta}(u)}{|\partial_u \boldsymbol{\gamma}(u)|}\, \mathrm{d}u \\
&= -\int_0^1 \partial_u \left(\frac{\partial_u \boldsymbol{\gamma}(u)}{|\partial_u \boldsymbol{\gamma}(u)|} \right) \cdot \boldsymbol{\zeta}(u)\, \mathrm{d}u \\
&= \int_0^{L(\boldsymbol{\gamma})} \kappa(s) \boldsymbol{N}(s) \cdot \boldsymbol{\zeta}(s)\, ds,
\end{aligned}$$

where s is the arc-length parameter, and the last equality follows from the Frenet formulas. In particular, if we restrict the direction of the perturbation to the normal direction, i.e., $\boldsymbol{\zeta}(s) = p(s)\boldsymbol{N}(s)$, we obtain the formula of the first variation as

$$\delta L(\Gamma) = \int_0^{L(\boldsymbol{\gamma})} \kappa(s) p(s)\, \mathrm{d}s.$$

The first variation of the interfacial energy

Let Ω be a bounded region in $\mathbb{R}^2$, Ω_0 be a smooth Jordan region contained in Ω and write its boundary as Γ. Define the interfacial energy by

$$E(\Omega_0) := \int_{\Omega \setminus \Omega_0} \gamma(\boldsymbol{x})\, \mathrm{d}\boldsymbol{x}.$$

Let $\boldsymbol{T}_\varepsilon \colon \Omega_0 \to \Omega_\varepsilon$, $\Omega_\varepsilon := \boldsymbol{T}_\varepsilon(\Omega_0)$, $0 \le \varepsilon \ll 1$, be a family of C^1-diffeomorphism such that $\boldsymbol{T}_0 = \mathrm{id}$. Let us compute the first variation of the interfacial energy $E(\Omega_0)$, referring to the paper[?]. In other words, consider

$$\begin{aligned}\left.\frac{\mathrm{d}}{\mathrm{d}\varepsilon}E(\Omega_\varepsilon)\right|_{\varepsilon=0} &= \left.\frac{\mathrm{d}}{\mathrm{d}\varepsilon}\int_{\Omega\setminus\Omega_\varepsilon}\gamma(\boldsymbol{x})\,\mathrm{d}\boldsymbol{x}\right|_{\varepsilon=0} \\ &= \left.\frac{\mathrm{d}}{\mathrm{d}\varepsilon}\int_{\Omega_0\setminus\boldsymbol{T}_\varepsilon(\Omega_0)}\gamma(\boldsymbol{x})\,\mathrm{d}\boldsymbol{x}\right|_{\varepsilon=0} \\ &= \left.\frac{\mathrm{d}}{\mathrm{d}\varepsilon}\int_{\Omega\setminus\Omega_0}\gamma(\boldsymbol{T}_\varepsilon(\boldsymbol{y}))\det J(\boldsymbol{T}_\varepsilon(\boldsymbol{y}))\,\mathrm{d}\boldsymbol{y}\right|_{\varepsilon=0} \\ &= \left.\int_{\Omega\setminus\Omega_0}\nabla\gamma(\boldsymbol{T}_\varepsilon(\boldsymbol{y}))\cdot\frac{\partial\boldsymbol{T}_\varepsilon}{\partial\varepsilon}(\boldsymbol{y}) + \gamma(\boldsymbol{T}_\varepsilon(\boldsymbol{y}))\frac{\partial}{\partial\varepsilon}\det J(\boldsymbol{T}_\varepsilon(\boldsymbol{y}))\,\mathrm{d}\boldsymbol{y}\right|_{\varepsilon=0},\end{aligned}$$

where $J(\boldsymbol{T}_\varepsilon(\boldsymbol{y}))$ denotes the Jacobian matrix of $\boldsymbol{T}_\varepsilon$ at $\boldsymbol{y}$. Since

$$\left.\frac{\partial}{\partial\varepsilon}\det J(\boldsymbol{T}_\varepsilon(\boldsymbol{y}))\right|_{\varepsilon=0} = \nabla\cdot\left.\frac{\partial\boldsymbol{T}_\varepsilon}{\partial\varepsilon}(\boldsymbol{y})\right|_{\varepsilon=0} = \nabla\cdot\boldsymbol{S}(\boldsymbol{y}),$$

we have

$$\left.\frac{\mathrm{d}}{\mathrm{d}\varepsilon}E(\Omega_\varepsilon)\right|_{\varepsilon=0} = \int_{\Omega\setminus\Omega_0}\left(\nabla\gamma(\boldsymbol{T}_0(\boldsymbol{y}))\cdot\boldsymbol{S}(\boldsymbol{y}) + \gamma(\boldsymbol{T}_0(\boldsymbol{y}))\nabla\cdot\boldsymbol{S}(\boldsymbol{y})\right)\mathrm{d}\boldsymbol{y},$$

where $\boldsymbol{S} := (\partial\boldsymbol{T}_\varepsilon/\partial\varepsilon)|_{\varepsilon=0}$ and we have used the relation $\boldsymbol{T}_0 = \mathrm{id}$. Restricting the perturbation to the normal direction as in the computation of the first variation of the length, we obtain $p(s) = \boldsymbol{S}(s)\boldsymbol{N}(s)$ on Γ. As a result, we have

$$\left.\frac{\mathrm{d}}{\mathrm{d}\varepsilon}E(\Omega_\varepsilon)\right|_{\varepsilon=0} = -\int_\Gamma \gamma(s,t)p(s,t)\,\mathrm{d}s,$$

that is,

$$\delta E(\Omega_0) = -\int_\Gamma \gamma(s,t)p(s,t)\,\mathrm{d}s.$$

The first variation of the area-preserving energy

As in the previous section, let Ω_0 be a smooth Jordan region and write its boundary as Γ. The area-preserving energy is defined by

$$S(\Omega_0) := \beta(|\Omega_0| - A)^2 = \beta\left(\int_{\Omega_0}\mathrm{d}\boldsymbol{x} - A\right)^2,$$

where A is the area of the self-propelled object at the initial time. This energy is minimised when the area of Ω_0 coincides with A.

Let $\boldsymbol{T}_\varepsilon \colon \Omega_0 \to \Omega_\varepsilon$, $\Omega_\varepsilon := \boldsymbol{T}_\varepsilon(\Omega_0)$, $0 \le \varepsilon \ll 1$, be a smooth family of C^1-diffeomorphism such that $\boldsymbol{T}_0 = \mathrm{id}$. Let us compute the first variation of the area-preserving energy $S(\Omega_0)$. A similar calculation as in the previous section yields

$$\begin{aligned}\left.\frac{\mathrm{d}}{\mathrm{d}\varepsilon}S(\Omega_\varepsilon)\right|_{\varepsilon=0} &= \left.\frac{\mathrm{d}}{\mathrm{d}\varepsilon}\beta\left(\int_{\Omega_\varepsilon}\mathrm{d}\boldsymbol{x} - A\right)^2\right|_{\varepsilon=0} \\ &= 2\beta\left(\int_{\Omega_\varepsilon}\mathrm{d}\boldsymbol{x} - A\right)\left.\frac{\mathrm{d}}{\mathrm{d}\varepsilon}\int_{\boldsymbol{T}_\varepsilon(\Omega_0)}\mathrm{d}\boldsymbol{x}\right|_{\varepsilon=0} \\ &= 2\beta\,(|\Omega_0| - A)\int_{\Omega_0}\nabla\cdot\boldsymbol{S}(\boldsymbol{y})\,\mathrm{d}\boldsymbol{y} \\ &= 2\beta\,(|\Omega_0| - A)\int_\Gamma \boldsymbol{S}(s)\cdot\boldsymbol{N}(s)\,\mathrm{d}s.\end{aligned}$$

Again, restricting the perturbation to the normal direction as in previous sections, we obtain

$$\frac{\mathrm{d}}{\mathrm{d}\varepsilon}S(\Omega_\varepsilon)\bigg|_{\varepsilon=0} = \int_\Gamma 2\beta(|\Omega_0|-A)p(s)\,\mathrm{d}s,$$

that is,

$$\delta S(\Omega_0) = \int_\Gamma 2\beta(|\Omega_0|-A)p(s)\,\mathrm{d}s.$$

Formal derivation of the singular-limit model in a two-dimensional case

In what follows, we demonstrate the interface equation by the formal argument.

Outer expansion

Assume that φ and u have the following expansions away from the interface $\Gamma(t)$:

$$\varphi = \varphi^0 + \varepsilon\varphi^1 + O(\varepsilon^2), \quad u = u^0 + \varepsilon u^1 + O(\varepsilon^2). \tag{S1}$$

Substituting the expansion (S1) into (3) and collecting the term ε^0, we know that φ^0 satisfies $\varphi^0(1-\varphi^0)(\varphi^0 - 1/2) = 0$, and hence

$$\varphi^0 = \begin{cases} 1, & \boldsymbol{x} \in \Omega^{\mathrm{in}}(t), \\ 0, & \boldsymbol{x} \notin \Omega^{\mathrm{in}}(t). \end{cases}$$

Similarly, u^0 satisfies

$$\frac{\partial u^0}{\partial t} = \triangle u^0 - ku^0 + \varphi^0, \quad \boldsymbol{x} \in \Omega \setminus \Gamma(t), \quad t > 0. \tag{S2}$$

Note that u is continuous if u^0 is continuous.

Inner expansion

We consider the formal expansion near $\Gamma(t)$. Let p be an arc length parameter of $\Gamma(t)$ (counter-clockwise) and q be a distance parameter along the normal direction at the point $\boldsymbol{x}_0(p) \in \Gamma(t)$. Remark that any point $\boldsymbol{x}$ in the neighborhood of $\Gamma(t)$ is uniquely represented by two parameters (p,q), that is, $\boldsymbol{x} = \boldsymbol{x}(p,q) = \boldsymbol{x}_0(p) + q\boldsymbol{\nu}(p)$, where $\boldsymbol{\nu}(p)$ is the outer normal unit vector at $\boldsymbol{x}_0(p)$. From this, two inverse functions $p = P(\boldsymbol{x},t)$ and $q = Q(\boldsymbol{x},t)$ for $\boldsymbol{x} \in \Gamma(t)$ are defined. Denote Φ and U by

$$\Phi(p,q,t) := \Phi(P(x,y,t), Q(x,y,t)/\varepsilon, t) = \varphi(\boldsymbol{x},t), \quad U(p,q,t) := U(P(x,y,t), Q(x,y,t)/\varepsilon, t) = u(\boldsymbol{x},t)$$

Substituting these two functions into (3), we have

$$\varepsilon^2\tau\left(\Phi_p P_t + \Phi_q\frac{Q_t}{\varepsilon} + \Phi_t\right) = \varepsilon^2\sigma^2\left(\Phi_{pp}|\nabla P|^2 + \Phi_p\triangle P + \frac{1}{\varepsilon^2}\Phi_{qq} + \frac{1}{\varepsilon}\Phi_q\triangle_p Q\right) + \Phi(1-\Phi)(\Phi - a(S[\varphi](t), U, \varepsilon)).$$

Here $\triangle_p$ stands for Laplace-Beltrami operator. We collect the ε^1 term and obtain the relation

$$\sigma^2\Phi_{qq} + f(\Phi) + \varepsilon(\sigma^2\kappa\Phi_q + \tau V\Phi_q + \Phi(1-\Phi)(\gamma(U) - S[\varphi](t))) + O(\varepsilon^2) = 0, \tag{S3}$$

where $f(\Phi) = \Phi(1-\Phi)(\Phi - 1/2)$, κ is the mean-curvature on $\Gamma(t)$, V is the normal velocity of $\Gamma(t)$. Assume that Φ and U have the following expansions near the interface $\Gamma(t)$:

$$\Phi = \Phi^0 + \varepsilon\Phi^1 + O(\varepsilon^2), \quad U = U^0 + \varepsilon U^1 + O(\varepsilon^2). \tag{S4}$$

Substituting (S4) into (S3), we have

$$\sigma^2\Phi^0_{qq} + f(\Phi^0) + \varepsilon((\sigma^2\kappa + \tau V)\Phi^0_q + \sigma^2\Phi^1_{qq} + f'(\Phi^0)\Phi^1 + \Phi^0(1-\Phi^0)(\gamma(U^0) - S[\varphi^0](t))) + O(\varepsilon^2) = 0,$$

where $S[\varphi^0](t) = \alpha(\int_\Omega \varphi^0(\boldsymbol{x},t)\,d\boldsymbol{x} - \int_\Omega \varphi^0(\boldsymbol{x},0)\,d\boldsymbol{x})$. Here we used the fact that $S[\varphi](t)$ is represented by

$$S[\varphi](t) = S[\varphi^0](t) + O(\varepsilon), \tag{S5}$$

Actually, (S5) is obtained by the explanation below. Let $\Omega_\varepsilon(t)$ be a tubular domain, including $\Gamma(t)$, in which the inner expansion is defined and matches the outer expansion. Let $\Omega_{\gamma_1}(t)$ and $\Omega_{\gamma_2}(t)$ be bounded regions with closed boundaries $\gamma_1(t;\varepsilon)$ and $\gamma_2(t;\varepsilon)$, respectively, on which the inner and outer expansions match. Without loss of generality, we can assume that

$$\Omega_{\gamma_1}(t) \subset \Omega_{\gamma_2}(t) \subset \Omega_\varepsilon(t)$$

Set $\Omega_{\gamma_1,\gamma_2}(t) := \Omega_{\gamma_1}(t) \setminus \Omega_{\gamma_2}(t) \neq \emptyset$. By (S1) and (S4),

$$\int_\Omega \varphi\,d\boldsymbol{x} = \int_{\Omega\setminus\Omega_{\gamma_1,\gamma_2}(t)} \varphi^0\,d\boldsymbol{x} + \int_{\Omega_{\gamma_1,\gamma_2}(t)} \Phi^0\,d\boldsymbol{x} + O(\varepsilon).$$

It follows from the assumption that $\Omega_\varepsilon(t)$ has to converge to the surface $\Gamma(t)$ and the Lebesgue measure $m(\Omega_{\gamma_1,\gamma_2}(t))$ goes to 0 as $\varepsilon \to 0$. By $|\varphi^0| \le 1$, we have

$$\int_{\Omega\setminus\Omega_{\gamma_1,\gamma_2}(t)} \varphi^0\,d\boldsymbol{x} = \int_\Omega \varphi^0\,d\boldsymbol{x} - \int_{\Omega_{\gamma_1,\gamma_2}(t)} \varphi^0\,d\boldsymbol{x} = \int_\Omega \varphi^0\,d\boldsymbol{x} + O(\varepsilon).$$

Similarly, Φ^0 is uniformly bounded and hence

$$\int_{\Omega_{\gamma_1,\gamma_2}(t)} \Phi^0\,d\boldsymbol{x} = O(\varepsilon).$$

As a result,

$$S[\varphi](t) = \alpha\left(\int_\Omega \varphi(\boldsymbol{x},t)\,d\boldsymbol{x} - \int_\Omega \varphi(\boldsymbol{x},0)\,d\boldsymbol{x}\right) = \alpha\left(\int_\Omega \varphi^0(\boldsymbol{x},t)\,d\boldsymbol{x} - \int_\Omega \varphi(\boldsymbol{x},0)\,d\boldsymbol{x}\right) + O(\varepsilon)$$

and then (S5) is concluded.

As for Φ, the following two equations are obtained :

$$\sigma^2\Phi^0_{qq} + f(\Phi^0) = 0, \tag{S6}$$

$$(\sigma^2\kappa + \tau V)\Phi^0_q + \sigma^2\Phi^1_{qq} + f'(\Phi^0)\Phi^1 + \Phi^0(1-\Phi^0)(\gamma(U^0) - S[\varphi^0](t)) = 0. \tag{S7}$$

Meanwhile, the equation with respect to U^0 is obtained as follows :

$$\varepsilon^{-2}d_u U^0_{qq} + \varepsilon^{-1}Q_t U^0_q + O(1) = 0. \tag{S8}$$

By the matched conditions of the inner solution and outer solution, U and Φ need to satisfy

$$\lim_{q\to-\infty}\Phi^0 = 0, \quad \lim_{q\to+\infty}\Phi^0 = 1, \tag{S9}$$

$$\lim_{q\to\pm\infty} U^0 = \lim_{q\to\pm 0} u^0(\boldsymbol{x}(p,q),t). \tag{S10}$$

According to (S8), (S10) and the continuity of u^0, it holds $U^0(p,q,t) \equiv u^0(\boldsymbol{x}(p,0),t)$, and then U^0 is independent of q. Since the function Φ^0 satisfies (S6) and (S9), it follows that

$$\Phi^0 = \frac{1}{1+e^{q/(\sqrt{2}\sigma)}}, \quad \sigma\Phi^0_q = -\frac{1}{\sqrt{2}}\Phi^0(1-\Phi^0)$$

and hence (S7) is rewritten by

$$\sigma^2\Phi^1_{qq} + f'(\Phi^0)\Phi^1 = (-(\sigma^2\kappa + \tau V) + \sqrt{2}\sigma(\gamma(U^0) - S[\varphi^0](t)))\Phi^0_q.$$

Differentiating both sides of (S6) in q, we have

$$\sigma^2(\Phi^0_q)_{qq} + f'(\Phi^0)\Phi^0_q = 0.$$

Set $L = \sigma^2 d^2/dq^2 + f'(\Phi^0)$, and then L is a self-adjoint operator (see Lemma2.2 in Ref.?). Hence

$$0 = (L\Phi^0_q, \Phi^1) = (\Phi^0_q, L\Phi^1) = (-(\sigma^2\kappa + \tau V) + \sqrt{2}\sigma(\gamma(U^0) - S[\varphi^0](t)))\|\Phi^0_q\|^2_{L^2(\mathbb{R}^2)}$$

and the following interface equation is obtained :

$$\tau V = -\sigma^2\kappa + \sqrt{2}\sigma(\gamma(U^0) - S[\varphi^0](t)). \tag{S11}$$

Combining (S2) and (S11), we obtain the free boundary problem (6).

A numerical scheme for interface model

This section summarises a numerical scheme for the interface model that is accurate second-order in space and time.

Regularisation of the characteristic function

The characteristic function χ appears in the second equation in the reaction-diffusion equation in the interface model, which is not continuous. We replace it with a smooth function obtained by adjusting Fourier coefficients.

Let $\hat{\chi}_\Omega(k,l)$ denote the two-dimensional Fourier coefficient of χ_Ω for each $k,l \in \mathbb{Z}$. The decay rate of the Fourier coefficients in the limit as $|k|,|l| \to \infty$ characterises the regularity of the function. Since χ_Ω is not smooth, the decay of its Fourier coefficients as $|k|,|l| \to \infty$ is very slow. We obtain a smooth approximation of the characteristic function by multiplying the Fourier coefficient by an exponentially decaying function. More precisely, let $0 < \eta \ll 1$ and define $\hat{\chi}^\eta_\Omega(k,l)$ by

$$\hat{\chi}^\eta_\Omega(k,l) := \hat{\chi}_\Omega(k,l)\exp\left(-\eta(k^2+l^2)\right), \quad k,l \in \mathbb{Z}.$$

Applying the inverse Fourier transform to $\left\{\hat{\chi}^\eta_\Omega(k,l)\right\}_{k,l}$ yields the smoothed characteristic function χ^ε_Ω.

In the following, we use the above-obtained smoothed characteristic function χ^η_Ω instead of χ_Ω.

Spatial discretisation

Approximation of the interface by a polygonal curve

We approximate the original smooth curve by a polygonal curve and compute its time evolution. Namely, suppose that Γ is represented as follows:

$$\Gamma = \bigcup_{i=1}^{N} \Gamma_i, \quad \Gamma_i = (\boldsymbol{X}_{i-1}, \boldsymbol{X}_i) := \{(1-\lambda)\boldsymbol{X}_{i-1} + \lambda\boldsymbol{X}_i \mid \lambda \in (0,1)\}.$$

Then, the time evolution of the interface is described by a polygonal time evolution law described by a system of ordinary differential equations:

$$\dot{\boldsymbol{X}}_i = V_i\boldsymbol{N}_i + W_i\boldsymbol{T}_i, \quad i = 1,2,\dots,N. \tag{S12}$$

Here, $\boldsymbol{T}_i$ and $\boldsymbol{N}_i$ denote the unit tangent vector and the unit outward normal vector of Γ at $\boldsymbol{X}_i$, respectively, defined in the subsequent subsubsection.

Unit tangent vector, unit outward normal vector, and discrete curvature

Denote the length of the ith edge Γ_i by $r_i = |\boldsymbol{X}_i - \boldsymbol{X}_{i-1}|$. The unit tangent vector $\boldsymbol{t}_i$ and the unit outward normal vector $\boldsymbol{n}_i$ on Γ_i are respectively defined as

$$\boldsymbol{t}_i = \frac{\boldsymbol{X}_i - \boldsymbol{X}_{i-1}}{r_i}, \quad \boldsymbol{n}_i = -\boldsymbol{t}_i^{\perp}, \quad i = 1, 2, \ldots, N,$$

where, for $\boldsymbol{a} = (a,b)^{\top} \in \mathbb{R}^2$, $\boldsymbol{a}^{\perp}$ denotes the vector obtained by rotating $\boldsymbol{a}$ by angle $\pi/2$; that is, $\boldsymbol{a}^{\perp} = (-b,a)^{\top}$. Let φ_i be the (signed) outer angle of Γ at $\boldsymbol{X}_i$, which can be computed by

$$\varphi_i = \operatorname{sgn}(D)\arccos(I), \quad D = \det\begin{pmatrix}\boldsymbol{t}_i & \boldsymbol{t}_{i+1}\end{pmatrix}, \quad I = \boldsymbol{t}_i \cdot \boldsymbol{t}_{i+1}.$$

Here, sgn is the signum function defined by

$$\operatorname{sgn}(x) = \begin{cases} 1 & \text{if } x > 0, \\ 0 & \text{if } x = 0, \\ -1 & \text{if } x < 0. \end{cases}$$

We define the unit tangent vector $\boldsymbol{T}_i$ at $\boldsymbol{X}_i$ as the unit vector parallel to and with the same direction as $\boldsymbol{t}_i + \boldsymbol{t}_{i+1}$. Since the length of $\boldsymbol{t}_i + \boldsymbol{t}_{i+1}$ can be computed as $2\cos(\varphi_i/2)$, we have

$$\boldsymbol{T}_i = \frac{\boldsymbol{t}_i + \boldsymbol{t}_{i+1}}{2\cos(\varphi_i/2)}, \quad i = 1, 2, \ldots, N.$$

We then define the unit outward normal vector $\boldsymbol{N}_i$ by $\boldsymbol{N}_i = -\boldsymbol{T}_i^{\perp}$.

The length $L[\Gamma]$ of Γ is

$$L[\Gamma] = \sum_{i=1}^{N} r_i.$$

Under the polygonal evolution law (S12), the time derivative $\partial_t \mathscr{L}[\Gamma]$ of $L[\Gamma]$ can be computed as

$$\partial_t L[\Gamma] = 2\sum_{i=1}^{N} V_i \sin\frac{\varphi_i}{2}. \tag{S13}$$

Let $\boldsymbol{X}_i^* = (\boldsymbol{X}_i + \boldsymbol{X}_{i-1})/2$ be the midpoint of Γ_i, and define the dual edge Γ_i^* by $\Gamma_i^* = (\boldsymbol{X}_i^*, \boldsymbol{X}_i) \cup (\boldsymbol{X}_i, \boldsymbol{X}_{i+1}^*)$. The length of Γ_i^* is $r_i^* = (r_i + r_{i+1})/2$. Then, we can rewrite (S13) as follows:

$$\partial_t L[\Gamma] = \sum_{i=1}^{N} \frac{2\sin(\varphi_i/2)}{r_i^*} V_i r_i^*.$$

Comparing this expression with

$$\partial_t L[\Gamma] = \int_{\Gamma} \kappa V \, \mathrm{d}s,$$

which is valid for smooth curves, we define the discrete curvature κ_i^* on Γ_i^* by

$$\kappa_i^* = \frac{2\sin(\varphi_i/2)}{r_i^*}, \quad i = 1, 2, \ldots, N.$$

Approximation of the Laplacian by central-finite differences

In order to compute the reaction-diffusion equation in the interface model, we introduce a uniform mesh

$$x_{1i} = -\frac{L_1}{2} + \left(i - \frac{1}{2}\right)\Delta x_1, \quad i = 1,2,\dots,N_1,$$
$$x_{2j} = -\frac{L_2}{2} + \left(j - \frac{1}{2}\right)\Delta x_2, \quad j = 1,2,\dots,N_2,$$

where $\Delta x_i := L_i/N_i$ for $i = 1,2$. Then, we approximate the Laplacian $\triangle$ by the central-finite differences; denoting an approximation of u at (x_{1i}, x_{2j}) by u_{ij}, we have

$$(\triangle u)(x_{1i}, x_{2j}) \approx \delta^2 u_{ij} := \frac{u_{i+1,j} - 2u_{ij} + u_{i-1,j}}{(\Delta x_1)^2} + \frac{u_{i,j+1} - 2u_{ij} + u_{i,j-1}}{(\Delta x_2)^2}.$$

In addition, the periodic boundary condition is imposed as follows:

$$u_{0j} = u_{N_1,j}, \quad u_{N_1+1,j} = u_{1j}, \quad j = 1,2,\dots,N_2,$$
$$u_{i0} = u_{i,N_2}, \quad u_{i,N_2+1} = u_{i1}, \quad i = 1,2,\dots,N_1.$$

As a result, the reaction-diffusion equation reduces to a system of ordinary differential equations:

$$\frac{\mathrm{d}u_{ij}}{\mathrm{d}t} = \delta^2 u_{ij} - k u_{ij} + \chi^{\varepsilon}_{\Omega^t}(x_{1i}, x_{2j}), \quad i = 1,2,\dots,N_1, \ j = 1,2,\dots,N_2.$$

Computation of the normal velocity

Normal velocities at vertices can be computed using the discrete curvature κ_i as

$$V_i = -\frac{\sigma^2}{\tau}\kappa_i^* + \frac{\sqrt{2}}{\tau}\gamma(u_{\mathrm{vert},i}) + \frac{\sqrt{2}}{\tau}\alpha\left(|\Omega^{\mathrm{in}}(0)| - |\Omega^{\mathrm{in}}(t)|\right),$$

where $u_{\mathrm{vert},i}$ denotes the value of u at $\boldsymbol{X}_i$, computed by the spline interpolation of $\{u_{ij}\}$.

Computation of the tangential velocity by the uniform distribution method

To perform stable numerical computation, we need to set tangential velocities appropriately. Among several choices, we adopt the asymptotic uniform distribution method.

Suppose that a set of real numbers $\{\eta_i\}_{i=1}^N$ satisfies

$$r_i - \frac{\mathscr{L}[\Gamma]}{N} = \eta_i \exp(-f(N,t)), \quad |\eta_i| \le 1, \quad \sum_{i=1}^N \eta_i = 0,$$

where $f(N,t)$ satisfies $\lim_{t\to T_{\max}} f(N,T) = \infty$. Here, $T_{\max}$ denotes a final computation time. One can also add the assumption $\lim_{N\to\infty} f(N,t) = \infty$. By differentiating the above relation for t, we have

$$\dot{r}_i - \frac{\partial_t L[\Gamma]}{N} = \left(\frac{L[\Gamma]}{N} - r_i\right)\omega(N,t),$$

where $\omega(N,t) = \partial_t f(N,t)$. On the other hand, $\dot{r}_i$ can be directly computed as

$$\dot{r}_i = (\dot{\boldsymbol{X}}_i - \dot{\boldsymbol{X}}_{i-1})\cdot \boldsymbol{t}_i = V_i \sin\frac{\varphi_i}{2} + V_{i-1}\sin\frac{\varphi_{i-1}}{2} + W_i\cos\frac{\varphi_i}{2} - W_{i-1}\cos\frac{\varphi_{i-1}}{2}.$$

Combining these two relations with a zero-average condition $\sum_{i=1}^N W_i = 0$, we obtain

$$W_1 = -\frac{\sum_{i=2}^N \Psi_i/\cos(\varphi_i/2)}{\cos(\varphi_1/2)\sum_{i=1}^N(\cos(\varphi_i/2))^{-1}}, \quad W_i = \frac{\Psi_i + W_1\cos(\varphi_1/2)}{\cos(\varphi_i/2)} \quad (i = 2,3,\dots,N),$$

where

$$\Psi_i = \sum_{j=2}^N \psi_j, \quad \psi_j = -V_j\sin\frac{\varphi_j}{2} - V_{j-1}\sin\frac{\varphi_{j-1}}{2} + \frac{\partial_t L[\Gamma]}{N} + \left(\frac{L[\Gamma]}{N} - r_j\right)\omega \quad (j = 2,3,\dots,N).$$

Temporal discretisation

We adopt the second-order explicit discretisation method in time.

Runge–Kutta method

In the first step only, we adopt the explicit second-order Runge–Kutta method, which offers the following discretisation

$$y^{n+1} = y^n + \Delta t f(t^{n+1/2}, y^{n+1/2}), \quad y^{n+1/2} = y^n + \frac{\Delta t}{2} f(t^n, y^n)$$

for the ordinary differential equation

$$\dot{y} = f(t, y).$$

Here, Δt denotes a (uniform) time increment, and $t^n := n\Delta t$.

Adams–Bashforth method

Except for the first step, we adopt the second-order Adams–Bashforth method:

$$y^{n+1} = y^n + \frac{3}{2}\Delta t f(t^n, y^n) - \frac{1}{2}\Delta t f(t^{n-1}, y^{n-1}).$$

Temporal discretisation procedure

We discretise our problem in time using the two temporal discretisation methods mentioned above. Denote approximations of u_{ij} and $\boldsymbol{X}_i$ at time t^n by u_{ij}^n at $\boldsymbol{X}_i^n$, respectively.

1. Set initial values u_{ij}^0 and $\boldsymbol{X}_i^0$.

2. Compute u_{ij}^1 and $\boldsymbol{X}^1$ using the explicit second-order Runge–Kutta method,

$$\begin{pmatrix} u_{ij}^1 \\ \boldsymbol{X}_l^1 \end{pmatrix} = \begin{pmatrix} u_{ij}^0 \\ \boldsymbol{X}_l^0 \end{pmatrix} + \Delta t \begin{pmatrix} d_u \delta^2 u_{ij}^{1/2} - k u_{ij}^{1/2} + k_0 s_0 \chi_{\Omega^{1/2}}^{\eta}(x_{1i}, x_{2j}) \\ V_l^{1/2} \boldsymbol{N}_l^{1/2} + W_l^{1/2} \boldsymbol{N}_l^{1/2} \end{pmatrix},$$

$$\begin{pmatrix} u_{ij}^{1/2} \\ \boldsymbol{X}_l^{1/2} \end{pmatrix} = \begin{pmatrix} u_{ij}^0 \\ \boldsymbol{X}_l^0 \end{pmatrix} + \frac{\Delta t}{2} \begin{pmatrix} d_u \delta^2 u_{ij}^0 - k u_{ij}^0 + k_0 s_0 \chi_{\Omega^0}^{\eta}(x_{1i}, x_{2j}) \\ V_l^0 \boldsymbol{N}_l^0 + W_l^0 \boldsymbol{N}_l^0 \end{pmatrix}.$$

3. For $n \geq 1$, compute u_{ij}^{n+1} and $\boldsymbol{X}_{ij}^{n+1}$ using the Adams–Bashforth method,

$$\begin{pmatrix} u_{ij}^{n+1} \\ \boldsymbol{X}_l^{n+1} \end{pmatrix} = \begin{pmatrix} u_{ij}^n \\ \boldsymbol{X}_l^n \end{pmatrix} + \frac{3\Delta t}{2} \begin{pmatrix} d_u \delta^2 u_{ij}^n - k u_{ij}^n + k_0 s_0 \chi_{\Omega^n}^{\eta}(x_{1i}, x_{2j}) \\ V_l^n \boldsymbol{N}_l^n + W_l^n \boldsymbol{T}_l^n \end{pmatrix} - \frac{\Delta t}{2} \begin{pmatrix} d_u \delta^2 u_{ij}^{n-1} - k u_{ij}^{n-1} + k_0 s_0 \chi_{\Omega^{n-1}}^{\eta}(x_{1i}, x_{2j}) \\ V_l^{n-1} \boldsymbol{N}_l^{n-1} + W_l^{n-1} \boldsymbol{T}_l^{n-1} \end{pmatrix}.$$
